# Proteomic profiling of retina and retinal pigment epithelium combined embryonic tissue to facilitate ocular disease gene discovery

**DOI:** 10.21203/rs.3.rs-2652395/v1

**Published:** 2023-03-17

**Authors:** Sandeep Aryal, Deepti Anand, Hongzhan Huang, Ashok P. Reddy, Phillip A. Wilmarth, Larry L. David, Salil A. Lachke

**Affiliations:** 1 Department of Biological Sciences, University of Delaware, Newark, DE 19716 USA; 2 Center for Bioinformatics & Computational Biology, University of Delaware, Newark, DE 19713 USA; 3 Proteomics Shared Resource, Oregon Health & Science University, Portland, OR 97239, USA; 4 Department of Chemical Physiology & Biochemistry, Oregon Health & Science University, Portland, OR 97239, USA

**Keywords:** Retina, Proteome, Mass spectrometry, Development, Embryogenesis, Gene discovery, iSyTE

## Abstract

To expedite gene discovery in eye development and its associated defects, we previously developed a bioinformatics resource-tool iSyTE (integrated Systems Tool for Eye gene discovery). However, iSyTE is presently limited to lens tissue and is predominantly based on transcriptomics datasets. Therefore, to extend iSyTE to other eye tissues on the proteome level, we performed high-throughput tandem mass spectrometry (MS/MS) on mouse embryonic day (E)14.5 retina and retinal pigment epithelium combined tissue and identified an average of 3,300 proteins per sample (n=5). High-throughput expression profiling-based gene discovery approaches-involving either transcriptomics or proteomics–pose a key challenge of prioritizing candidates from thousands of RNA/proteins expressed. To address this, we used MS/MS proteome data from mouse whole embryonic body (WB) as a reference dataset and performed comparative analysis-termed “*in silico* WB-subtraction”–with the retina proteome dataset. *In silico* WB-subtraction identified 90 high-priority proteins with retina-enriched expression at stringency criteria of ≥2.5 average spectral counts, ≥2.0 fold-enrichment, False Discovery Rate <0.01. These top candidates represent a pool of retina-enriched proteins, several of which are associated with retinal biology and/or defects (*e.g.*, Aldh1a1, Ank2, Ank3, Dcn, Dync2h1, Egfr, Ephb2, Fbln5, Fbn2, Hras, Igf2bp1, Msi1, Rbp1, Rlbp1, Tenm3, Yap1, *etc.*), indicating the effectiveness of this approach. Importantly, *in silico* WB-subtraction also identified several new high-priority candidates with potential regulatory function in retina development. Finally, proteins exhibiting expression or enriched-expression in the retina are made accessible in a user-friendly manner at iSyTE (https://research.bioinformatics.udel.edu/iSyTE/), to allow effective visualization of this information and facilitate eye gene discovery.

## Introduction

Identification of genes associated with ocular development and its associated defects remains a challenge even though there has been widespread application of transcriptomics and proteomics toward these goals in recent years (Anand and Lachke 2017; Ahmad et al. 2018; Wolf et al. 2022). This is because high-throughput approaches such as RNA-sequencing or mass spectrometry identify thousands of candidates (RNA or protein) and the principal challenge lies in the application of effective downstream analyses for prioritizing candidates that are relevant to the development, homeostasis or pathology of the specific tissue/cell type of interest. Furthermore, prioritization strategies based solely on the extent of expression (*i.e.*, absolute expression) may result in missing important regulatory candidates that are not necessarily among the most highly expressed, but may nevertheless have a key role in development of the tissue/cell type. We developed a user-friendly web-based resource-tool called iSyTE (integrated Systems Tool for Eye gene discovery) to address these challenges in the eye; however, the earlier versions were focused on the lens (Lachke et al. 2012b; Kakrana et al. 2018; Anand et al. 2018; Aryal et al. 2020a). In addition to absolute expression in a tissue/cell type, iSyTE prioritizes candidate genes by comparing the gene expression profiles – in this case, of the lens – to that of a reference dataset, namely, mouse embryonic whole body (WB), a process termed “*in silico* WB-subtraction”. This results in prioritization of candidates based on their “enriched-expression” in the lens as opposed to solely based on their absolute expression. Application of the iSyTE approach has effectively identified many new genes (*e.g.*, *Caprin2*, *Celf1*, *Mafg*, *Mafk*, *Rbm24*, *Tdrd7*, etc.) as well as led to the characterization of several regulatory pathways associated with eye/lens development and defects (Lachke et al. 2011, 2012a, b; Kasaikina et al. 2011; Wolf et al. 2013; Manthey et al. 2014; Agrawal et al. 2015; Dash et al. 2015, 2020; Audette et al. 2016; Patel et al. 2017, 2022; Anand and Lachke 2017; Cavalheiro et al. 2017; Siddam et al. 2018; Krall et al. 2018; Padula et al. 2019; Barnum et al. 2020; Aryal et al. 2020b; Anand et al. 2021; Choquet et al. 2021; Lachke 2022).

The present version of iSyTE is restricted to data on the lens, and furthermore, it is predominantly based on transcriptomic data in the form of microarrays or RNA-seq (Lachke et al. 2012b; Kakrana et al. 2018; Anand et al. 2018), with limited data on the proteome (Aryal et al. 2020a). Moreover, it is known that the correlation between RNA profiles and protein profiles is complex and not necessarily linear (Maier et al. 2009). Contributing to this is regulation at the post-transcriptional level – that involves non-coding RNA or RNA-binding protein (RBP)-mediated control over mRNA splicing, transport, stability and translation – all of which determines the cellular proteome (Brinegar and Cooper 2016; Hentze et al. 2018; Gebauer et al. 2021). Indeed, recent findings have demonstrated that RBPs play a conserved role in mediating post-transcriptional control in eye and lens development (Lachke et al. 2011; Lachke and Maas 2011; Dash et al. 2015, 2016, 2020; Siddam et al. 2018; Shao et al. 2020; Barnum et al. 2020; Nakazawa et al. 2020; Sundar et al. 2020; Aryal et al. 2020b; Lachke 2022; Matalkah et al. 2022). Together, these findings suggest that along with the transcriptome, characterization of the proteome is important to determine the factors that are important in a specific cell/tissue of interest. While there are currently many independent transcriptomics studies on the retina (Ratnapriya et al. 2019; Clark et al. 2019; Lukowski et al. 2019; Liang et al. 2019), studies on the retina proteome, especially in embryonic development, are limited (Zhao et al. 2007; Mizukami et al. 2008; Finnegan et al. 2008; Balasubramani et al. 2010). To address these knowledge-gaps in the context of eye development, we report here a tandem mass spectrometry (MS/MS)-based protein profiling of the mouse embryonic day (E) 14.5 retina and retinal pigment epithelium (RPE) combined (termed henceforth as retina) and its comparative analysis with *in silico* WB-subtraction. We demonstrate that while retina protein expression alone (*i.e.*, retina proteome not subjected to *in silico* subtraction) can identify several genes linked to retina biology and defects and is in itself helpful, *in silico* WB-subtraction provides another effective approach in prioritizing key candidates that are not necessarily among the highest expressed proteins in the retina. We generated new expression tracks at the University of California at Santa Cruz (UCSC) Genome Browser and make this new data accessible through iSyTE.

## Methods

### Animals

Mice of the background C57BL/6J, obtained from The Jackson Laboratory, were used as wild-type animals in this study, and were bred and maintained at the University of Delaware Center for Animal research facility. The Institutional Animal Care and Use Committee (IACUC) approved the animal protocol (AUP#1226). All the animal experiments described in this study were performed in adherence to the guidelines in the Association of Research in Vision and Ophthalmology (ARVO) statement for the use of animals in ophthalmic and vision research.

### Tissue Collection

Mice were bred and pregnant females were euthanized for obtaining embryos for collection of retina tissue. The day on which a vaginal plug was detected was designated as embryonic day (E) 0.5, and tissues was collected at E14.5. Whole retina tissue (retina + retinal pigment epithelium (RPE), henceforth referred to as “retina”) were micro-dissected from E14.5 mouse embryos and stored at −80°C until further processing. Whole embryonic tissue minus the eye at E14.5 was considered as “whole embryonic body” (WB). Five biological replicates with each replicate consisting of two retinas isolated from the same embryo were collected. Tissues were processed as previously described (Aryal et al. 2020a). Briefly, tissue samples were suspended in 120 μl of TEAB buffer (167 mM triethyl ammonium bicarbonate buffer) and subjected to probe-sonication in a Fisher Scientific 60 Sonic Dismembrator. To these lysed samples, 40 μl of 20% SDS, 1% DCA and 40 μl of water were added to bring the total volume of each sample to 200 μl (final concentration: 4% SDS, 0.2% DCA, 100 mM TEAB), which were next centrifuged (16000 x *g*, 2 min., room temperature) followed by heating (90°C for 15 min.). Sample protein quantification was estimated by BCA protein assay kit (Thermo Fisher Cat. No. 23225). For each biological replicate (*n* = 5 biological replicates), 55 μg of protein/sample was subjected to trypsinization as previously described (Erde et al. 2017). Briefly, a modified enhanced filter-aided digestion protocol (e-FASP) using Amicon 30 kDa ultracentrifugation devices was executed. Samples were subjected to TCEP (Tris Carboxy Ethyl Phosphene) reducing reagent at 90°C for 10 min, followed by transferring to an Amicon filter. Samples were then buffer exchanged into 8 M Urea, 0.2% deoxycholic acid (DCA), 100 mM TEAB. Next, samples were subjected to alkylation with iodoacetamide, exchanged into 0.2% DCA, 50 mM TEAB (pH 8.0) digestion buffer, and subjected to overnight digestion by trypsin (1:20 enzyme:substrate concentration). After overnight trypsin digestion, samples were subjected to centrifugation and the filtrate, which contained the peptides, was subjected to extraction with ethyl acetate, which served to remove DCA. A SpeedVac vacuum concentrator (Thermo Fisher Scientific) was then used to dry the samples which were then resuspended in 100 μl of HPLC-grade water. Next, a Pierce Quantitative Colorimetric Peptide Assay Kit was used to perform a peptide assay on the samples and the average peptide recovery from mouse E14.5 retina samples was estimated to be ~45 μg/sample. Whole embryonic body (WB) tissue (eye removed) sample processing was performed as previously described (Aryal et al. 2020a).

### Mass Spectrometry

Mass spectrometry (MS) was performed as previously described (Aryal et al. 2020a). Briefly, protein samples (concentration: 4 μg in 5% Formic acid) were loaded for 5 min on an Acclaim PepMap 0.1 x 20 mm NanoViper C18 peptide trap (Thermo Fisher Scientific)(flow rate: 10 μl/min; mobile phase: in a 2% acetonitrile, 0.1% formic acid). PepMap RSLC C18 2 μm particle, 75 μm x 50 cm EasySpray column (Thermo Fisher Scientific) was used for separating peptides over 205 min on a 7.5–30% acetonitrile gradient (mobile phase: 0.1% formic acid, 300 nl/min flow rate) with Dionex NCS-3500RS UltiMate RSLC nano UPLC system. An Orbitrap Fusion mass spectrometer configured with an EasySpray NanoSource (Thermo Fisher Scientific) was used to collect tandem MS data, using data dependent analysis (DDA) configuration and a MS/DD-MS/MS instrument menthod (full MS resolutions: 120,000 at m/z 200, mass range 375-1500, charge state 2-7; full MS AGC target: 400,000; intensity threshold: 5,000; max inject time: 50 ms and 10 ppm dynamic exclusion for 60 s; AGC target value for fragment spectra: 5,000; isolation mode: quadrupole; isolation width: 1.6 m/z; isolation offset: off; activation type: CID; collision energy: fixed 35%; maximum injection time: 300 ms; detector type: IonTrap). Centroid mode using positive polarity was used to acquire data. The mass spectrometry proteomics data have been deposited to the ProteomeXchange Consortium via the Proteomics IDEntifications (PRIDE) database (https://www.ebi.ac.uk/pride/) (Perez-Riverol et al. 2022) partner repository with the dataset identifier PXD039490.

### Raw File Conversion and Database Search

MSConvert (Proteowizard toolkit) was used to convert RAW files to MS2 format for the samples as described (Chambers et al. 2012; Aryal et al. 2020a). The retina samples had ~74K MS2 scans per run. A software (available at https://github.com/pwilmart/fasta_utilities.git) was used to download a canonical mouse reference proteome (version 2019.04; 22,287 sequences) from UniProt. To this, a concatenated sequence-reversed decoy database was added along with common contaminants (179 sequences) to obtain 44,932 entries. Peptide sequences were assigned to the MS2 spectra (PSMs) using the search engine Comet (Eng et al. 2013), which was configured as previously described (Aryal et al. 2020a) and were as follows: tryptic cleavage (maximum of two missed cleavages); monoisotopic parent ion mass tolerance of 1.25 Da; monoisotopic fragment ion tolerance of 1.0005 Da; fragment bin offset of 0.4; b-, y-, and neutral loss ions were used in scoring (flanking peaks were not used); variable modification of oxidation (+15.9949 Da) on methionine was specified; static modification of alkylation (+57.0215 Da) of cysteines was specified.

### PSM Error Control

The PAW pipeline (https://github.com/pwilmart/PAW_pipeline.git) and the target/decoy method described previously (Elias and Gygi 2007; Wilmarth et al. 2009) was used for post-processing the highest scoring matches for individual PSMs (obtained from Comet) using false discovery rate (FDR) error control. Peptides of different charge states (2+, 3+, and 4+ were considered) and modification state (unmodified or oxidized) were processed to derive accurate delta mass conditional score histograms. FDR values were estimated based on target and decoy score histograms as a function of a Peptide-Prophet-like discriminant score to set thresholds for experiment-wide PSM FDR of 1% as described previously (Keller et al. 2002; Aryal et al. 2020a). A minimum length of 7 amino acids-length were considered for peptide matches. The number of confidently identified (1% FDR) PSMs per sample was 35.4K and the identification rate was 48%.

### Protein Inference

The expressed proteins were inferred, using basic parsimony principles, based on the filtered PSM sequences (Nesvizhskii and Aebersold 2005). Homologous protein family members were grouped using an extended parsimony algorithm when evidence to distinguish family members was insufficient. In total, 3,963 proteins were detected after grouping (excluding common contaminant proteins) with 37 decoy matches, for a protein FDR of about 0.9%. The average number of proteins identified per sample was 3,296.

### Quantitative Analysis

For the retina and the WB samples, equal amounts of protein were digested and the total spectral counts (SpC, a robust semi-quantitative measure) were measured. Prior to protein inference, the SpC for individual samples were tallied and they independently validated the peptide assay results. Next, the retina and the WB samples were matched by subjecting the individual samples to be scaled to the average total spectral count per sample. Both the retina and the WB samples had about 3,300 protein identifications per sample. Next, the proteins with enriched expression in the retina compared to WB were determined as follows: For individual proteins, the average SpC for all samples was computed from the scaled data, and only values greater than 2.5 (2,675 proteins) were considered in the differential expressed enrichment analysis between the retina and WB. The bioconductor package, edgeR, was used for differential gene expression analysis (Robinson and Oshlack 2010; Robinson et al. 2010). The default Benjamini-Hochberg multiple testing corrections and the exact test in edgeR were applied in R (version 3.5.3).

### Gene Ontology Analysis

For functional annotation by gene ontology (GO) categories, a cluster-based analysis using the Database for Annotation, Visualization and Integrated Discovery (DAVID v6 .8) (Huang et al. 2009) was performed on candidate proteins with retina-expression and retina-enriched expression that were identified by *in silico* WB-subtraction (the cut-offs were: ≥2.5 average spectral counts, ≥2.0 fold-enrichment, FDR <0.01). Benjamini corrected significant *p*-values were considered for prioritization of the pathways and GO categories identified from this analysis as previously described (Aryal et al. 2020a).

## Results and Discussion

### Embryonic retina proteome generation and quality assessment

**Fig. 1.**
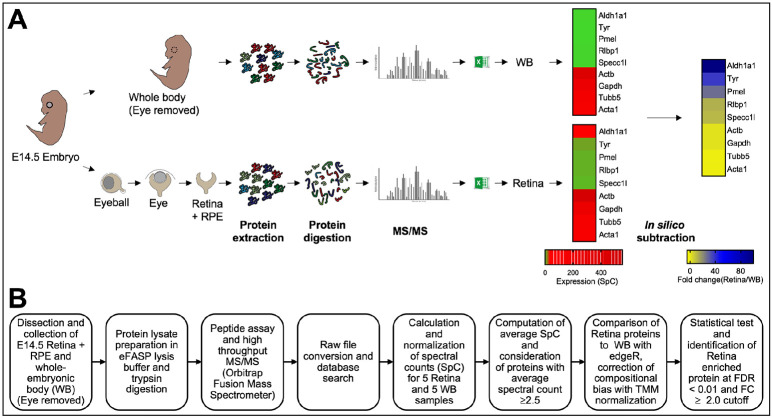
Workflow of the experimental strategy to generate MS/MS protein profile of the mouse embryonic retina and retinal pigment epithelium combined tissue. (A) Mouse eyes at embryonic day (E)14.5 were isolated, and the retina and retinal pigment epithelium combined tissue (termed retina) was micro-dissected. The whole body (WB) with eye tissue removed was processed similarly and used as reference for differential protein expression analysis. Retina and WB samples (*n* = 5 for each sample type, 55 μg protein per sample) were subjected to high-throughput tandem mass spectrometry (MS/MS). (B) The workflow for differential protein expression analysis is outlined. The edgeR pipeline was used to determine differential protein expression using normalized spectral counts. Proteins passing stringency criteria of ≥2.5 average spectral counts, ≥2.0 fold-change (in retina, compared to WB), False Discovery Rate <0.01 were considered to have enriched expression in the retina.

**Fig. 2.**
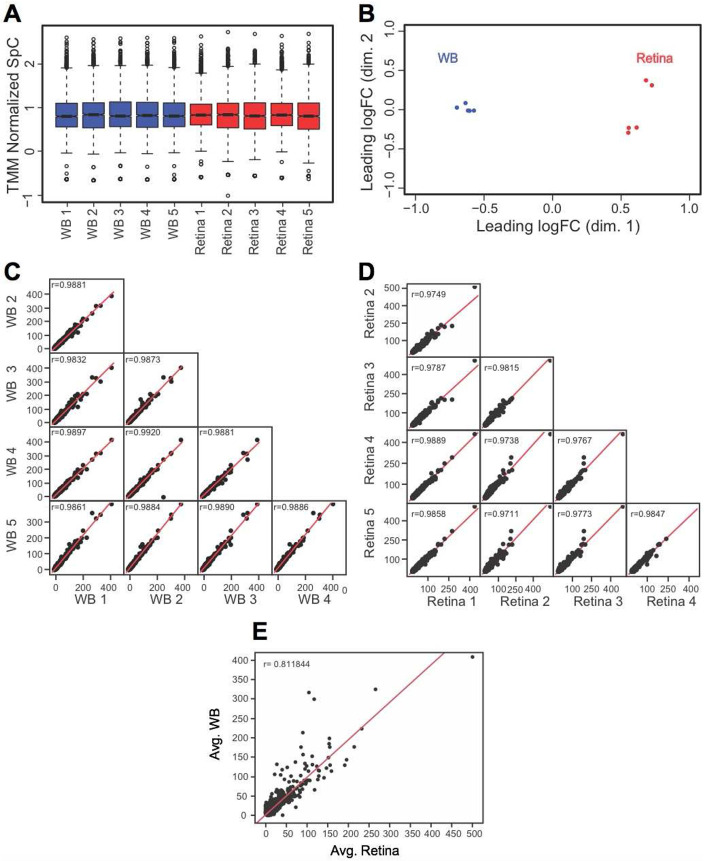
Quality assessment of MS/MS data. (A) TMM (trimmed mean of *M*-values) normalization of spectral counts in WB and retina samples using edgeR to correct for the dramatic compositional differences. The retina and the WB samples showed comparable median SpCs in boxplots (TMM normalized SpC are shown in *y*-axis). (B) Individual biological replicates of the retina and WB samples clustered together while the overall retina and WB samples clustered separately from each other in Multidimensional scaling analysis (leading dimensions 1 and 2 are represented by the axes). (C) Sample-to-sample consistency was examined by generating a scatter matrix for the five WB samples and (D) the five retina samples. (E) A scatter plot with regression analysis shows no correlation *(r* = 0.81) between the average retina and average WB samples.

We designed an experimental workflow to isolate mouse E14.5 retina, generate its proteome and perform *in silico* WB-subtraction (Fig. 1A). Retina tissue was micro-dissected from mouse E14.5 eyes and processed for protein preparation and proteome analysis. Mouse WB preparation was performed as previously described (Aryal et al. 2020a). Further, proteome downstream analyses were performed according to the outlined workflow (Fig. 1B). From individual retina and WB samples (*n* = 5 biological replicates), 55 μg of protein were subjected to eFASP (enhanced filter-aided sample preparation) and digestion by trypsin. After digestion, equal amounts of peptides were used for high-throughput tandem mass spectrometry (MS/MS) and spectral count (SpC) data were generated. Application of stringent criteria (≥2 distinct peptides per protein in at least one sample, ≥2.5 average SpC in the retina) to the resulting data led to enrichment analysis of 2,675 proteins in the E14.5 retina (Supplementary Table S1). Across the samples, on average ~35K SpC were detected. Total average SpC was subjected to TMM (trimmed mean of M-values) normalization using edgeR package (Robinson et al. 2010) to account for differences in SpC between retina and WB (Fig. 2A). Next, the quality of data was assessed by boxplots for the normalized SpC datasets that demonstrated that the median expression levels were similar between all the retina and the WB samples (Fig. 2A). Further, multidimensional scaling-based cluster analysis was performed to examine the quality of TMM normalized SpC proteome data. Cluster analysis demonstrated that all five biological replicates of the retina clustered closer to each other and distinctly away from WB samples, which themselves clustered together (Fig. 2B). Sample to sample correlation within the retina and within the WB samples was examined by scatter plot comparisons in all combinations for retina and WB samples, which demonstrated high correlation between samples of the same type (Fig. 2C, D). While the five WB samples correlated with each other at *r* value >0.98, all the five retina samples correlated with each other at *r* value >0.97. Finally, comparing the correlation between the average SpC of the retina and that of WB shows that the correlation is much lower (*r* = 0.81) between the retina and WB (Fig. 2E).

### Gene Ontology (GO) analysis of proteins expressed in the E14.5 mouse

Before performing other downstream analysis, we characterized the E14.5 proteins that were identified. Many proteins previously linked to retinal development and disease were identified in the proteome analysis, based solely on expression (Supplementary Table S1 for all proteins and Supplementary Table S2 for the top 150). To examine whether specific pathways relevant to retina biology were enriched in this dataset, a cluster-based analysis was performed using the Database for Annotation, Visualization and Integrated Discovery (DAVID v6 .8) for functional annotation by gene ontology (GO) categories.

**Fig. 3.**
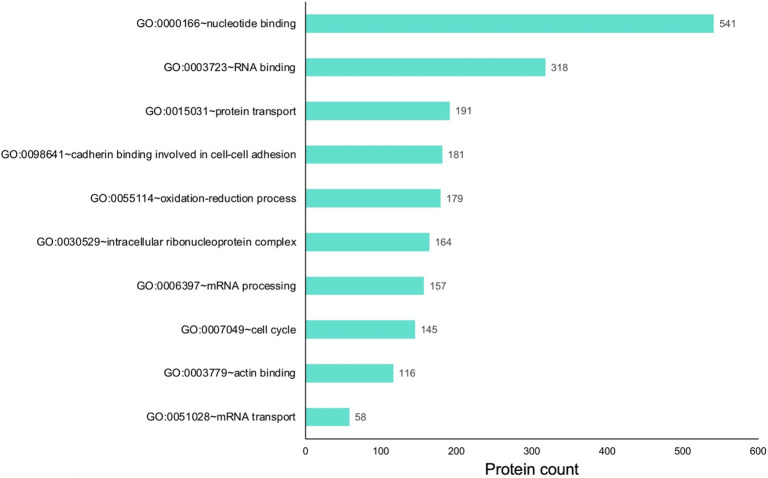
Gene ontology (GO) analysis of proteins expressed in the E14.5 retina and retinal pigment epithelium combined tissue. Proteins expressed in the retina were subjected to cluster-based analysis using the Database for Annotation, Visualization, and Integrated Discovery (DAVID v6 .8) for functional annotation by gene ontology (GO) categories. This analysis identified candidates representing several GO terms that may be relevant to retinal biology, including those involved in various molecular, cellular and physiological processes. The *x*-axis represents the number of protein candidates identified in the specific GO term shown on the *y*-axis.

This analysis identified several interesting pathways. These were related to post-transcriptional control of gene expression, *e.g.*, “GO:0003723 RNA-binding”, “GO:0030529 intracellular ribonucleoprotein complex”, “GO:0006397 mRNA processing” and “GO:0051028 mRNA transport” (Fig. 3)(Supplementary Table S3). Proteins involved in other molecular pathways and processes *e.g.*, “GO:0015031 protein transport”, “GO:0055114 oxidation-reduction process” were also identified in the dataset. Finally, pathways in basic cell biological processes were also enriched, *e.g.*, “GO:0007049 cell cycle”, “GO:0098641 cadherin binding involved in cell-cell adhesion”, and “GO:0003779 actin binding” in the total proteins expressed in E14.5 mouse retina. Together, these represent promising new candidates for future investigations in the retina.

### MS/MS *in silico* WB-subtraction identifies proteins exhibiting retina-enriched expression

**Fig. 4.**
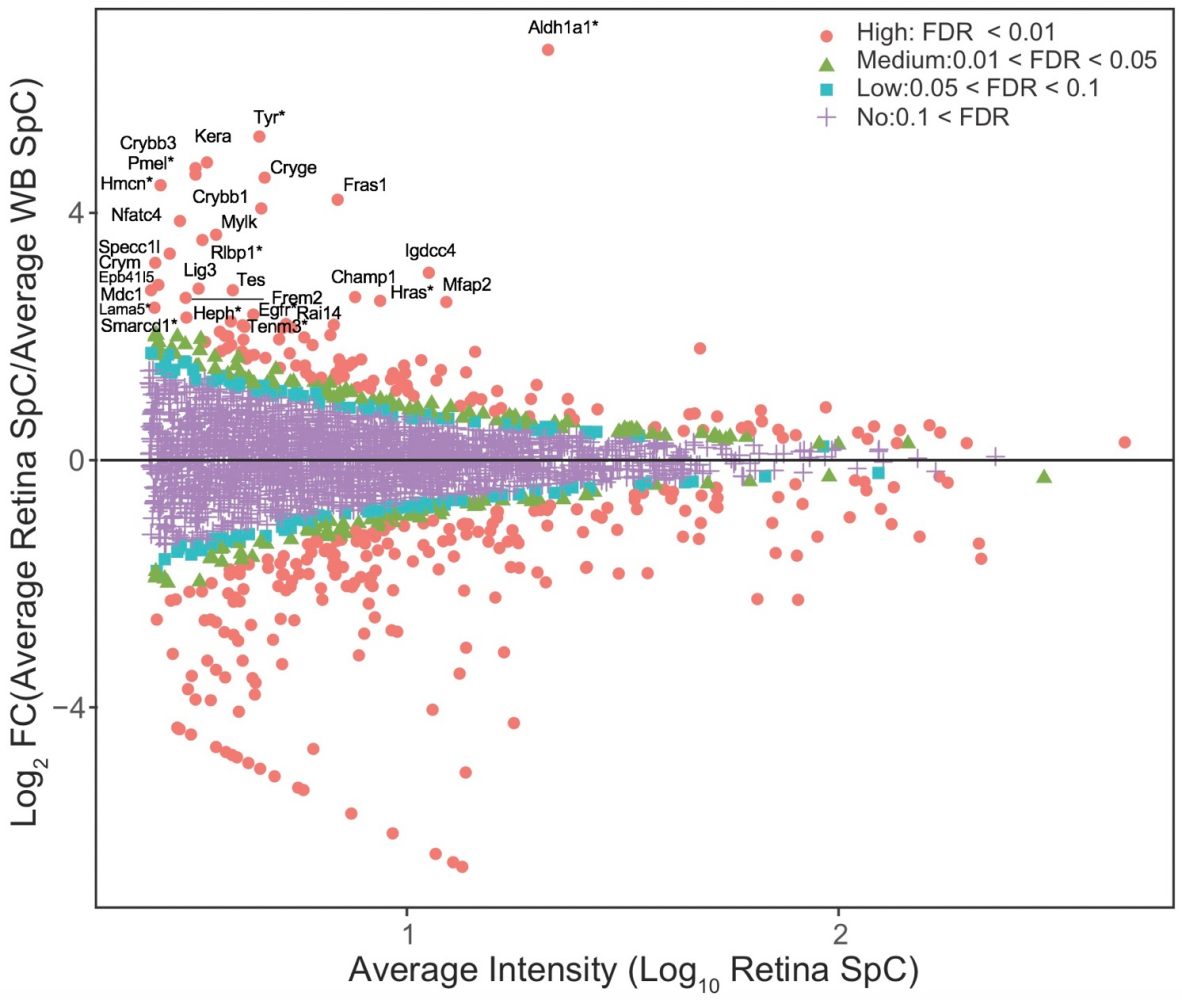
*In silico* WB-subtraction identifies candidates with enriched expression in the mouse embryonic retina and retinal pigment epithelium combined tissue. (A) Proteins with the average SpC ≥ 2.5 between retina and WB samples (*n*=2675) were further processed for identifying differentially expressed candidates. This analysis showed that 90 proteins were enriched in retina compared to WB ( ≥ 2.0 fold-change, FDR <0.01 cut-off). (B) MA plot (M = log ratio of retina to WB, A = average intensity) representation of differential protein expression profiling wherein the “high” (red, circle, FDR < 0.01), “medium” (green, triangle, 0.01 < FDR < 0.05), “low” probability retina-enriched (blue, square, 0.05 < FDR < 0.1) and non-enriched candidates (magenta, cross, 0.1 < FDR) are indicated. Several candidates associated with retinal defects (*) can be identified in this plot.

**Fig. 5.**
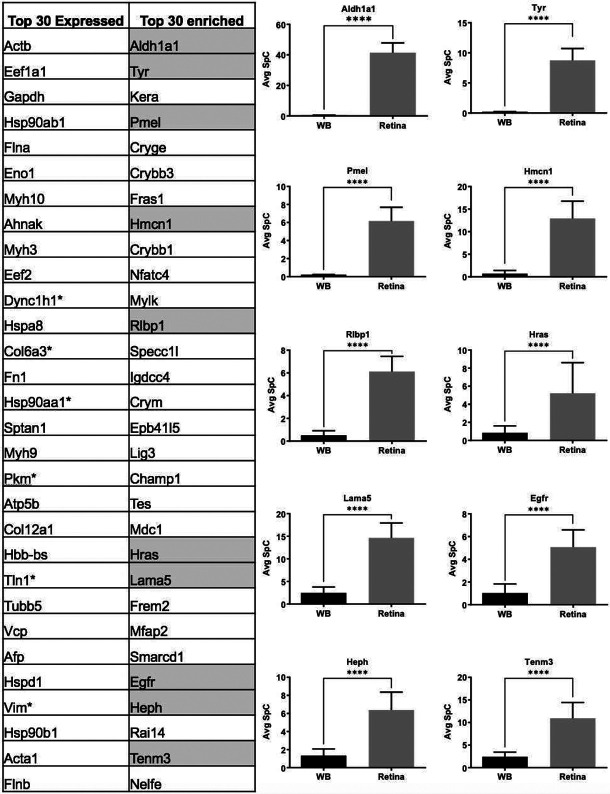
Prioritization of candidates associated with retina defects by *in silico* WB-subtraction. (A) Comparison of top 30 retina expressed proteins with the top 30 retina “enriched-expression” proteins shows that 1/3^rd^ of the candidates in the “enriched-expression” category are associated with the retinal defects (highlighted in grey) and are not amongst the top proteins expressed in the retina. (B) Aldh1a1, Tyr, Pmel, Hmcn1, Rlbp1, Hras, Lama5, Egfr, Heph and Tenm3 associated with retinal defects that were not present in the top 30 “expressed” candidates show significant (*p*<0.001) enrichment in the retina compared to WB, demonstrating the effectiveness of the *in silico* WB-subtraction strategy in identifying these candidates from 2,675 expressed proteins. The average SpC of individual proteins are shown in *y*-axis.

While GO analysis of total expressed proteins were helpful, to further prioritize the candidates from the E14.5 retina proteome, the “*in silico* WB-subtraction” approach, which has been effectively applied for prioritizing cataract-linked genes in the lens, was applied to this dataset. To do so, we computed the average SpC for all samples and scaled (normalized) data for each protein. Those peptides that passed the filtration criteria of ≥2.5 SpC were considered in the analysis. This approach identified 2675 proteins that could be tested for differential expression between the retina and WB samples. At ≥2.0 fold-enrichment and FDR <0.01 cut-off, 90 proteins had enriched expression in the retina compared to WB (Table 1). These “retina-enriched” proteins identified many proteins linked to retinal defects and revealed several new promising candidates (Fig. 4) demonstrating that the *in silico* WB-subtraction approach can be effectively applied to the retina. Further, compared to absolute expression of proteins, *in silico* WB-subtraction could more effectively prioritize key proteins associated with retina biology and disease. For example, the top 30 proteins ranked on relative abundance in the retina (*i.e.*, not subjected to *in silico* WB-subtraction) did not contain a single protein that has been associated with retina development or defects/disease (Fig. 5A). Indeed, candidates in this list, termed “retina expression” list, were representative of general housekeeping/structural proteins such as Glyceraldehyde-3-phosphate dehydrogenase (Gapdh), Actins (Acta1, Actb), Myosins (Myh3, Myh9, Myh10), Tubulin (Tubb5), Collagen (Col12a1) and several others, not exclusively associated with retina biology. In sharp contrast, the list of the top 30 candidates identified by *in silico* WB-subtraction, termed “retina enriched” list of candidates, contained 1/3^rd^ (10 out of 30) candidates that have been associated with retinal biology and/or defects/disease (Fig. 5B). These are Aldehyde dehydrogenase family 1, subfamily A1 (Aldh1a1), Tyrosinase (Tyr), Keratocan (Kera), Melanocyte protein PMEL (Pmel), Hemicentin-1 (Hmcn1), Retinaldehyde-binding protein 1 (Rlbp1), Harvey rat sarcoma virus oncogene (Hras), Laminin subunit alpha-5 (Lama5), Epidermal growth factor receptor (Egfr), Hephaestin (Heph) and Teneurin-3 (Tenm3). Importantly, the top candidates identified by the *in silico* WB-subtraction approach contained regulatory proteins that are not necessarily among the top highly expressed proteins in the retina. In contrast, no regulatory proteins were detected in the top 30 retina expression list. Finally, the significant differences in SpC levels for different proteins between the retina and WB serves to explain the basis for the effectiveness of the *in silico* WB-subtraction strategy in prioritization of candidates relevant to the retina and its associated defects (Fig. 5B).

### Biological and disease relevance of top retina-enriched proteins

Next, we conducted a detailed analysis of all 90 retina-enriched candidates in the context of the published literature to determine their potential relevance to retina biology and defects. Application of this evidence-based curation identified 27 of the 90 (~30%) retina-enriched proteins prioritized by the *in silico* WB-subtraction strategy to be associated with retina biology and/or defects (Table 1). The topmost enriched gene Aldehyde dehydrogenase family 1, subfamily A1 (Aldh1a1; also known as Raldh1 (Retinal dehydrogenase 1)) is shown to regulate dorsal choroidal vasculature development via Sox9 upregulation in retinal pigmented epithelium in mice (Goto et al. 2018). The enriched-expression list also independently identified Tyrosinase (Tyr) protein which is essential for melanin biosynthesis and therefore critical for RPE (retinal pigment cells) and other retinal cells (Jeffery et al. 1994, 1997). Among the candidates is the premelanosome (Pmel) protein, whose deficiency in mice results in cell shape changes, *e.g.*, the normally “oblong” shaped melanosomes turn spherical in RPE cells (Hellström et al. 2011). Further, whole exome sequencing of patients with early-onset age-related macular degeneration (AMD) revealed a single base deletion in another candidate with retina-enriched expression, the hemicentin-1 (HMCN1) gene (Pras et al. 2015).

Several proteins involved in signaling pathways were identified among the candidates with retina-enriched expression. Mutations in the candidate, Harvey rat sarcoma virus oncogene (Hras), are associated with retinal dystrophy in two patients with Costello syndrome (Pierpont et al. 2017). Another factor prioritized by *in silico*-WB subtraction is the epidermal growth factor receptor (Egfr), which is associated with retinal cell fate determination (Lillien 1995). Additionally, eph receptor B2 (Ephb2) was identified and its deletion in mouse is associated with axonal degeneration in retinal ganglion cell (Fu and Sretavan 2012). The receptor-type tyrosine-protein phosphatase F (Ptprf) is known to be expressed in retinal ganglion cells in mice (Lorber et al. 2005). Further, mutation in Fibulin-5 (FBLN5) is reported in human patients with AMD (Stone et al. 2004; Lotery et al. 2006). Morpholino-based reduction in the retinol-binding protein 1 (Rbp1) has been shown to result in misfolding of outer segments of retina in *Xenopus* (Wang et al. 2010). Recessive mutations in another top candidate, retinaldehyde-binding protein 1 (RLBP1), cause Retinitis punctatta albescens in humans (Morimura et al. 1999).

Some RNA-binding proteins (RBPs) involved in post-transcriptional gene expression control that are linked to retina development and differentiation were also among the prioritized proteins. Musashi homolog 1 (Msi1) is an RBP whose deficiency causes photoreceptor morphogenesis defect in mice (Sundar et al. 2020). Another RBP, the insulin-like growth factor 2 mRNA-binding protein 1 (Igf2bp1) is required for retinal ganglion cell axon outgrowth in zebrafish (Gaynes et al. 2015a). Mex3 RNA-binding family member A (Mex3a) is expressed in the ciliary marginal zone in zebrafish (Naef et al. 2020). The mRNA cap guanine-N7 methyltransferase (Rnmt) is known to be expressed in *Xenopus* retina (Lokapally et al. 2016). Among other proteins with a regulatory function that were identified, deletion in mouse of the transcriptional coactivator Yes-associated protein 1 (Yap1) shows that Yap1 is essential for maintaining retinal pigmented epithelium differentiation (Lu et al. 2020).

Several proteins associated with the cell membrane and/or cytoskeleton were identified. Patients with mutation in the gap junction alpha-1 protein (Gja1) exhibit optic nerve and retinal dysplasia (Gabriel et al. 2011). The ankyrin proteins identified here, ankyrin-2 (Ank2) and ankyrin-3 (Ank3), have been shown to be essential for development of rod photoreceptors in mice (Kizhatil et al. 2009b, a)(Kizhatil et al., 2009a, 2009b)(Kizhatil et al., 2009a, 2009b)(Kizhatil et al., 2009a). Knockout mice for another candidate, the cell adhesion molecule 1 (Cadm1), exhibited impaired response to light stimulation and for structural integrity of rod synapses (Ribic et al. 2014). Deficiency of the transmembrane protein, teneurin-3 (Tenm3) in zebrafish causes abnormal retinal ganglion cell morphology and the lack of Tenm3 in mice lead to defects in binocular visual coordination (Leamey et al. 2007; Antinucci et al. 2013).

Similarly, extracellular proteins as well as other cellular proteins with relevance to retinal biology were identified. Genetic variants in fibrillin-2 C-terminal peptide (FBN2) are reported to contribute to AMD (Ratnapriya et al. 2014). Cytoplasmic dynein 2 heavy chain 1 (Dync2h1) mutations in humans have been associated with non-syndromic inherited retinal disease (Vig et al. 2020). Deficiency in mice of the macrophage migration inhibitory factor (Mif) is associated with reduction in proliferation and inhibition of preretinal angiogenesis (Wang et al. 2017). Further, deficiency in mice of the small leucine-rich proteoglycan family protein Decorin (Dcn) results in structural and microvascular defects in retina (Lim et al. 2018). Finally, deficiency of rootletin (Crocc), recognized as a major component of the ciliary rootlet, is reported to cause retinal degeneration in mice (Yang et al. 2005).

Some genes that function in early retina development were also identified. For example, the transcriptional repressor CTCF (Ctcf) was among these candidates, and its deletion in mouse forebrain at E8.5 causes apoptosis and reduced retinal tissue by E13.5 (Watson et al. 2014). Further, a point mutation identified in the mouse Laminin subunit alpha-5 (Lama5) showed defective retinal cup morphology as early as at E15.5 (Jones et al. 2020). Proteins involved in homeostasis were also identified in this study. For example, hephaestin (Heph) is reported to be essential for iron homeostasis in mice and its deficiency is associated with retinal degeneration (Hahn et al. 2004).

Several other proteins were identified that are independently reported to be expressed in retina, but their function has not been examined in detail. We identified several crystallins (Crybb1, Crybb3, Cryge, and Crym) that are known to be expressed in the retina and have been associated with retinal ganglion cell survival and regeneration (Xi et al. 2003; Piri et al. 2013). Interestingly, knockout mice for another crystallin prioritized in this study, alpha-crystallin A chain (Cryaa), exhibit retinal neovascularization defect (Xu et al. 2015). Similarly, a binding partner (NEDD8 ultimate buster 1 (NUB1)) of the retinal defect-associated protein, Aryl hydrocarbon receptor-interacting protein-like 1 (AIPL1), identified here, is shown to be expressed in developing and adult human retina (Akey et al. 2002). The non-histone chromosomal protein, HMG-14 (Hmgn1), is expressed throughout retina in adult mouse (Lucey et al. 2008). The multi-functional serine and arginine-rich (SR) and desmosome associated protein Pinin (Pnn) is independently reported to be expressed photoreceptors of developing mouse retina (Leu and Ouyang 2006).

A few other proteins identified here have been reported to be associated with eye defects or disease, but their specific function in the retina has not been explored in detail. For example, FRAS1-related extracellular matrix protein 2 (Frem2) is associated with Cryptophthalmos (Yu et al. 2018). Interestingly, keratocan (Kera) deficiency in mice is associated with corneal defects, but its role in the retina has not been examined (Liu et al. 2003). Finally, kinesin-like protein 1A (Kif1a) has been associated with optic nerve hyperplasia but its mechanistic role is not known in detail (Raffa et al. 2017).

Together, this documented association to retina biology of nearly 1/3^rd^ of the top 90 proteins identified by *in silico*-WB subtraction, renders confidence that other candidates may also have key roles in the retina and may be linked to its defects.

### Gene Ontology analysis of retina-enriched proteins

**Fig. 6.**
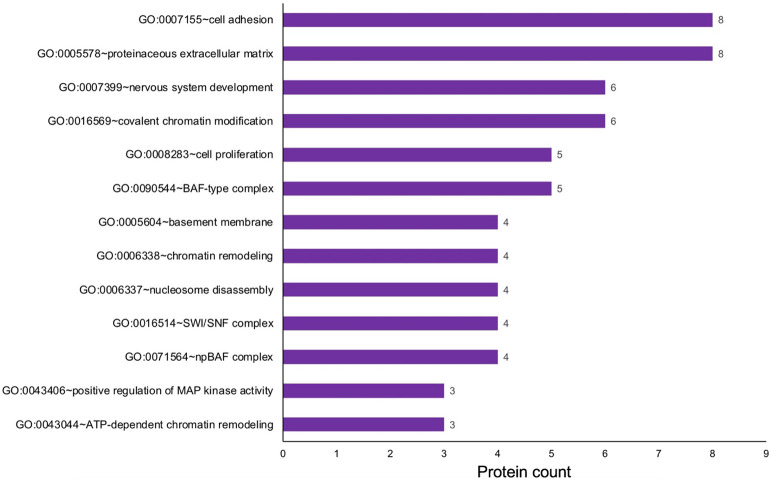
Gene ontology (GO) analysis of proteins with enriched expression in the E14.5 retina and retinal pigment epithelium combined tissue. The 90 proteins identified to exhibit “enriched expression” in the mouse retina and retinal pigment epithelium combined tissue were analyzed by the Database for Annotation, Visualization and Integrated Discovery (DAVID v6 .8) for functional clustering and annotation based on gene ontology (GO) categories. This analysis identified candidates representing several GO terms that are relevant to retina biology, including “nervous system development”, “positive regulation of MAP kinase activity”, “chromatin remodeling” and “cell adhesion”. The *x*-axis represent the number of protein candidates identified in the specific GO term shown on the *y*-axis.

To gain insights into the relevance of the 90 candidates identified by *in silico* WB-subtraction to retina biology, a cluster-based analysis was performed using the Database for Annotation, Visualization and Integrated Discovery (DAVID v6 .8) for functional annotation by gene ontology (GO) categories (Fig. 6) (Supplementary Table S4). This analysis assigned 90 retina enriched proteins into several annotation clusters. These are proteins involved in regulatory processes such as chromatin remodeling, *e.g.*, “GO:0006338 chromatin remodeling,” “GO:0016569 covalent chromatin modification”, “GO:0071564 npBAF complex”, “GO:0016514 SWI/SNF complex”, “GO:0090544 BAF-type complex”, “GO:0071565 nBAF complex”, “GO:0006337 nucleosome disassembly”, “GO:0043044 ATP-dependent chromatin remodeling”, as well as those involved in signaling pathways, *e.g.*, “GO:0043406 positive regulation of MAP kinase activity”. Proteins involved in basic cellular processes, *e.g.*, “GO:0008283 cell proliferation”, “GO:0007155 cell adhesion” were also identified. Additionally, proteins involved in extracellular matrix were identified, *e.g.*, “GO:0005604 basement membrane”, “GO:0005578 proteinaceous extracellular matrix”. Finally, proteins with roles in nervous system development were identified (Fig. 6) (Supplementary Table S4). Thus, this analysis identifies key candidates in specific processes relevant to retina biology, which can be functionally characterized in future studies.

### Visualization and access of retina-enriched and retina-expressed proteins in iSyTE

**Fig. 7.**
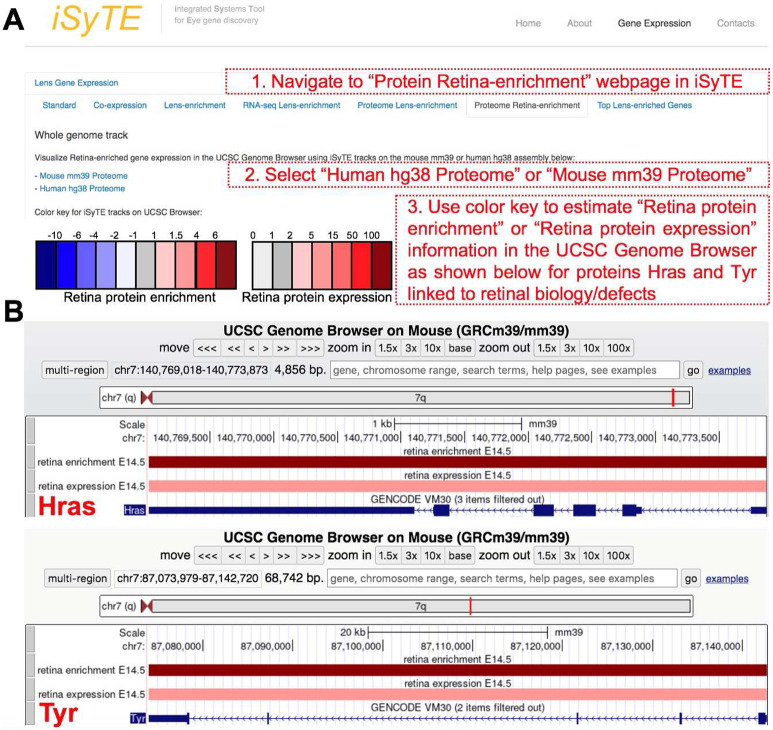
iSyTE allows ready visualization of proteins expressed or enriched-expressed in the retina and retinal pigment epithelium combined tissue. The retina protein expression and enriched-expression data can be visualized through the iSyTE web-resource tool at https://research.bioinformatics.udel.edu/iSyTE/. (A) On the iSyTE main webpage, navigate to “Proteome Retina-enrichment”, select the Human hg38 or Mouse mm39 assembly on the UCSC Genome Browser using iSyTE tracks, and input the protein candidate of interest to visualize its expression or enriched expression in the retina. The heat-map color key can be used to estimate the retina protein expression or enriched-expression. (B) As example, visualization of the expression and enriched expression of Hras and Tyr proteins in the retina are shown.

Next, we wanted to make this rich proteome information freely available to the research community. Thus, we developed new custom annotation-tracks on the UCSC Genome Browser that provide a heat-map representation of proteins based on their absolute expression or enriched-expression in the E14.5 mouse retina. These tracks are publicly accessible through the web-based resource-tool iSyTE (https://research.bioinformatics.udel.edu/iSyTE/). As examples, the enrichment and expression in the retina, of proteins previously linked to retina defects, *e.g.*, Hras and Tyr, are shown as visualized in iSyTE (Fig. 7A, B). This web-based resource-tool will allow ready and user-friendly visualization of proteins in the E14.5 mouse retina.

## Conclusion

Recent studies have highlighted that post-transcriptional regulation of gene expression plays a key role in determining the cellular proteome in eye development. Therefore, it is important to include ocular proteome data to the existing RNA-based profiling datasets to gain new insights into eye development. As a proof-of-principal we previously generated proteomic profiles for the mouse lens and the embryonic whole body (WB) and effectively applied *in silico* WB-subtraction strategy to identify proteins with lens-enriched abundance, which – in addition to consideration of absolute expression scores – allows a prioritized list of proteins for further study. In the present study, we expanded this approach to the mouse embryonic retina. We identified 90 proteins with retina-enriched expression. Nearly 1/3^rd^ of these candidates have been previously reported to be associated with retinal defects. This suggests that *in silico* WB-subtraction was effective in prioritizing select candidates from over 2,600 identified proteins and the other 2/3^rd^ of these identified proteins represent an unexplored pool of candidates for future characterization of their function in the retina. Indeed, there exist independent evidence in the literature for several of these candidates to be expressed in the retina, in agreement with the proteome data reported in the present study. Further, in addition to these “retina-enriched” candidates, nearly 4,000 proteins were found to be present in the mouse E14.5 retina proteome. It should be noted that while many proteins linked to retina biology and pathology were identified in this study, transcription factors (TFs) such as Otx2, Sox2 and Vsx2 with key roles in the retina were not detected. This may be due to the following reasons. While they may be enriched in tissues, TFs are often in lower abundance compared to other expressed proteins (Tacheny et al. 2013). Furthermore, their levels are often spatiotemporally restricted in specific cells within the tissue, information that is compromised when using bulk tissue (as is the case in the present study). In the present study, we measured static protein relative abundances and did not attempt dynamic system measurements (*e.g.*, those informing on protein turnover). Although 2675 quantifiable proteins (from the total 4680 proteins detected, which is generally considered a deep proteome) were identified in the present study, since the above mentioned TFs were not among these proteins, this suggests that more sensitive methods would be needed to detect these proteins in future studies. Together, these datasets and their ready accessibility through the web-based ocular gene discovery tool iSyTE represent a rich resource for prioritizing candidates for future hypothesis-driven studies in retina development. Finally, this study serves as a proof of the principle that *in silico* subtraction can also be applied to the retina and RPE to identify promising new candidates in these tissues. In the future, this approach will be expanded to prioritize candidates in other developmental stages of the retina.

## Figures and Tables

**Table 1.** Top 90 proteins with enriched expression in mouse E14.5 retina and retinal pigment epithelium combined tissue as compared to WB

Rank	UniProt Gene Name	Uniprot Accession	Primary Protein Name	Associated Retina or Eye Defects	Reference
1	Aldh1a1	P24549	Retinal dehydrogenase 1 (RALDH 1; RalDH1)	Expressed throughout development in dorsal retina; Choroidal hypoplasia in mice*	(Fan et al. 2003; Goto et al. 2018; Duester 2022)
2	Tyr	P11344	Tyrosinase	Retinal defects*	(Jeffery et al. 1994, 1997)
3	Kera	O35367	Keratocan (KTN)	-	
4	Pmel	Q60696	Premelanosome protein	RPE menalonosme cellular defect*	(Hellström et al. 2011)
5	Cryge	Q03740	Gamma-crystallin E	-	
6	Crybb3	Q9JJU9	Beta-crystallin B3, N-terminally processed	-	
7	Fras1	Q80T14	Extracellular matrix protein FRAS1	-	
8	Hmcn1	D3YXG0	Hemicentin-1	AMD*	(Pras et al. 2015)
9	Crybb1	Q9WVJ5	Beta-crystallin B1B	-	
10	Nfatc4	Q8K120	Nuclear factor of activated T-cells, cytoplasmic 4 (NF-ATc4; NFATc4)	-	
11	Mylk	Q6PDN3	Myosin light chain kinase, smooth muscle, deglutamylated form	-	
12	Rlbp1	Q9Z275	Retinaldehyde-binding protein 1	Retinitis punctatta albescens*	(Morimura et al. 1999)
13	Specc1l	Q2KN98	Cytospin-A	-	
14	Igdcc4	Q9EQS9	Immunoglobulin superfamily DCC subclass member 4	-	
15	Crym	O54983	Ketimine reductase mucrystallin	-	
16	Epb41l5	Q8BGS1	Band 4.1-like protein 5	-	
17	Lig3	P97386	DNA ligase 3	-	
18	Champ1	Q8K327	Chromosome alignment-maintaining phosphoprotein 1	-	
19	Tes	P47226	Testin	-	
20	Mdc1	Q5PSV9	Mediator of DNA damage checkpoint 1	-	
21	Hras	Q61411	GTPase HRas, N-terminally processed	Retinal dystrophy*	(Pierpont et al. 2017)
22	Lama5	Q61001	Laminin subunit alpha-5	Retinal defects*	(Jones et al. 2020)
23	Frem2	Q6NVD0	FRAS1-related extracellular matrix protein 2	Detected in outer plexiform layer of the retina; mutations associated with Cryptophthalmos	(Zhang et al. 2019)
24	Mfap2	P55002	Microfibrillar-associated protein 2 (MFAP-2)	-	
25	Smarcd1	Q61466	SWI/SNF-related matrix-associated actin-dependent regulator of chromatin subfamily D member 1	-	
26	Egfr	Q01279	Epidermal growth factor receptor	Retinal cell fate determination*	(Lillien 1995)
27	Heph	Q9Z0Z4	Hephaestin	Retinal defects*	(Hahn et al. 2004)
28	Rai14	Q9EP71	Ankycorbin	-	
29	Tenm3	Q9WTS6	Teneurin-3 (Ten-3)	Retinal defect in mouse and zebrafish*	(Leamey et al. 2007) (Antinucci et al. 2013)
30	Nelfe	P19426	Negative elongation factor E (NELF-E)	-	
31	Znf326	O88291	Zinc finger protein 326	-	
32	Crocc	Q8CJ40	Rootletin	Retinal defects*	(Yang et al. 2005)
33	Cadm1	Q8R5M8	Cell adhesion molecule 1	Retinal defects*	(Ribic et al. 2014)
34	Fbln5	Q9WVH9	Fibulin-5 (FIBL-5)	Retinal defects*	(Lotery et al. 2006) (Stone et al. 2004)
35	Wiz	O88286	Widely-interspaced zinc finger motifs	-	
36	Cryaa	P24622	Alpha-crystallin A chain	Retinal neovascularization defect*	(Xu et al. 2015)
37	Znf219	Q6IQX8	Zinc finger protein 219	-	
38	Gldc	Q91W43	Glycine dehydrogenase (decarboxylating), mitochondrial	-	
39	Mex3a	G3UYU0	Mex3 RNA-binding family member A	-	
40	Nub1	P54729	NEDD8 ultimate buster 1	-	
41	Uncharacterized protein FLJ45252 homolog	Q6PIU9	Uncharacterized protein FLJ45252 homolog	-	
42	Kif1a	P33173	Kinesin-like protein KIF1A	-	
43	Dync2h1	Q45VK7	Cytoplasmic dynein 2 heavy chain 1	Retinal defects*	(Vig et al. 2020)
44	Gja1	P23242	Gap junction alpha-1 protein	Retinal and other eye defects*	(Gabriel et al. 2011)
45	Fbn2	Q61555	Fibrillin-2 C-terminal peptide	AMD*	(Ratnapriya et al. 2014)
46	Pcca	Q91ZA3	Propionyl-CoA carboxylase alpha chain, mitochondrial (PCCase subunit alpha)	-	
47	Zmym4	A2A791	Zinc finger MYM-type protein 4	-	
48	Emilin3	P59900	EMILIN-3	-	
49	Tbl1x	Q9QXE7	Transducin (beta)-like 1 X-linked	-	
50	Bcat1	P24288	Branched-chain-amino-acid aminotransferase, cytosolic (BCAT(c))	-	
51	Niban1	Q3UW53	Protein Niban 1	-	
52	Ehmt2	Q9Z148	Histone-lysine N-methyltransferase EHMT2	-	
53	Brd7	O88665	Bromodomain-containing protein 7	-	
54	Taf15	Q8BQ46	TAF15 RNA polymerase II, TATA box binding protein (TBP)-associated factor	-	
55	Hspa4l	P48722	Heat shock 70 kDa protein 4L	-	
56	Msi1	Q61474	RNA-binding protein Musashi homolog 1 (Musashi-1)	Retinal defects*	(Sundar et al. 2020)
57	Aldh5a1	Q8BWF0	Succinate-semialdehyde dehydrogenase, mitochondrial	-	
58	Strn	O55106	Striatin	-	
59	Dido1	Q8C9B9	Death-inducer obliterator 1 (DIO-1)	-	
60	Ctcf	Q61164	Transcriptional repressor CTCF	Retinal defects*	(Watson et al. 2014)
61	Sptbn2	Q68FG2	Spectrin beta chain	-	
62	Rnmt	Q9D0L8	mRNA cap guanine-N7 methyltransferase	-	
63	Cux1	P53564	Homeobox protein cut-like 1	-	
64	Ephb2	P54763	EphB2/CTF2	Retinal defects*	(Fu and Sretavan 2012)
65	Golga5	Q9QYE6	Golgin subfamily A member 5	-	
66	Golgb1	E9PVZ8	Golgi autoantigen, golgin subfamily b, macrogolgin 1	-	
67	Ank2	Q8C8R3	Ankyrin-2 (ANK-2)	Retinal defects*	(Kizhatil et al. 2009b)
68	Snx6	Q6P8X1	Sorting nexin-6, N-terminally processed	-	
69	Srsf2	Q62093	Serine/arginine-rich splicing factor 2	-	
70	Smarce1	O54941	SWI/SNF-related matrix-associated actin-dependent regulator of chromatin subfamily E member 1	-	
71	Hmgn1	P18608	Non-histone chromosomal protein HMG-14	-	
72	Ptprf	A2A8L5	Receptor-type tyrosine-protein phosphatase F	-	
73	Dnajc7	Q9QYI3	DnaJ homolog subfamily C member 7	-	
74	Pnn	O35691	Pinin	-	
75	Mccc2	Q3ULD5	Methylcrotonoyl-CoA carboxylase beta chain, mitochondrial (MCCase subunit beta)	-	
76	Nipbl	Q6KCD5	Nipped-B-like protein	-	
77	Ank3	G5E8K5	Ankyrin-3 (ANK-3)	Retinal defects*	(Kizhatil et al. 2009a)
78	Rbp1	Q00915	Retinol-binding protein 1	Retinal defects*	(Wang et al. 2010)
79	Yap1	P46938	Transcriptional coactivator YAP1 (Yes-associated protein 1)	Retinal defects*	(Lu et al. 2020)
80	Smarcc1	P97496	SWI/SNF complex subunit SMARCC1	-	
81	Utrn	E9Q6R7	Utrophin	-	
82	Cald1	E9QA15	Caldesmon 1	-	
83	Ccar1	Q8CH18	Cell division cycle and apoptosis regulator protein 1	-	
84	Cttn	Q60598	Src substrate cortactin	-	
85	Prrc2c	Q3TLH4	Protein PRRC2C	-	
86	Arid1a	A2BH40	AT-rich interactive domain-containing protein 1A (ARID domain-containing protein 1A)	-	
87	Cbx1	P83917	Chromobox protein homolog 1	-	
88	Dcn	P28654	Decorin	Retinal defects*	(Lim et al. 2018)
89	Mif	P34884	Macrophage migration inhibitory factor (MIF)	Associated with proliferative retinopathy*	(Wang et al. 2017)
90	Igf2bp1	O88477	Insulin-like growth factor 2 mRNA-binding protein 1 (IGF2 mRNA-binding protein 1; IMP-1)	Retinal defects in Zebrafish*	(Gaynes et al. 2015b, p. 1)
